# Meniscus suture provides better clinical and biomechanical results at 1-year follow-up than meniscectomy

**DOI:** 10.1007/s00402-013-1681-1

**Published:** 2013-01-31

**Authors:** Juliusz Huber, Przemysław Lisiński, Paulina Kłoskowska, Agnieszka Gronek, Ewa Lisiewicz, Tomasz Trzeciak

**Affiliations:** 1 Department of Pathophysiology of Locomotor Organs, Wiktor Dega Clinical Orthopaedic and Rehabilitation Hospital, Karol Marcinkowski University of Medical Sciences, 28 Czerwca 1956r. No 135/147, 61-545 Poznań, Poland; 2 Department and Clinic for Physiotherapy, Rheumatology and Rehabilitation, Karol Marcinkowski University of Medical Sciences, Poznan, Poland; 3 Department and Clinic of Traumatology, Orthopedics and Hand Surgery, Karol Marcinkowski University of Medical Sciences, Poznań, Poland; 4 Department and Clinic of Orthopedics and Traumatology, Karol Marcinkowski University of Medical Sciences, Poznań, Poland

**Keywords:** Meniscectomy, Meniscus suture, Peak torque, One-leg-hop and one-leg-rising tests, H/Q coefficient, Supervised rehabilitation

## Abstract

**Background:**

Surgery of meniscus tear results in limitation of function. The aim of study was functional assessment of knee 1 year after surgery with two techniques in cases of the medial meniscus tear followed by the same supervised rehabilitation.

**Materials and methods:**

A total of 30 patients with good KOSS scores constituted two equal groups after partial meniscectomy or meniscus suture. Measurements of knee extensors and flexors muscles peak torques were performed with angular velocities 60, 180, 240 and 300 s^−1^ using Biodex IV system. One-leg-hop and one-leg-rising tests ascertained the function of operated knee. Results of examinations were compared with reference to healthy volunteers. Results of biomechanical and clinical studies were correlated to create complex and objective method evaluating treatment.

**Results:**

Extensors peak torque values at 60 s^−1^ angular velocity and H/Q coefficient were decreased after meniscectomy more than meniscus suture in comparison to healthy volunteers (*P* ≤ 0.001; *P* ≤ 0.05). Analysis of functional tests revealed that patients after meniscectomy showed difference between operated and non-operated knee (*P* ≤ 0.01) while patients with meniscus suture differed the least to controls (*P* ≤ 0.05). Extensors peak torque values at 60 s^−1^ angular velocity correlated with results of one-leg-rising test.

**Conclusion:**

Results suggest worse functional effects when meniscectomy is applied which implies modification of the rehabilitative methods in a postoperative period.

## Introduction

Partial resection of the injured meniscus, its suture or re-fixation are commonly applied in cases of the knee meniscus tear [1–4]. Partial meniscectomy is performed more frequently but results in significant functional deficits, mainly the decrease of muscle force in knee extensors [3, 5]. Proper knee stability and normal distribution of loadings between the joint’s surfaces significantly depend on the muscle force of thigh [3, 5, 6]. Hurley [7] confirmed the existence of early osteoarthritis after partial meniscectomy as a consequence of the thigh muscles force deficits [8]. The meniscus suturing protects the joint cartilage which in general prevents the early development of degenerative changes [9].

Deficits of the thigh muscle force can be evoked by mechanical injuries of meniscus proprioceptors accompanied to its suturing or simply by their deficits in cases of the partial meniscectomy [1, 10–13]. This was confirmed in studies on patients after meniscectomy using the surface electromyography [14]. This method indirectly allows for assessment of the motor units recruitment pattern which is created by activity of spinal and supraspinal centres receiving inputs from the proprioceptive afferents of knee joint including the meniscus receptors. This concept of multimodal coordination was also confirmed by Glatthorn et al. [15], who indicated on disturbances in a number and/or frequency of quadriceps muscle motor unit recruitment in patients following the partial meniscectomy. Akima et al. [1] performed muscle functional magnetic resonance imaging (mfMRI) examinations of muscles which results did not confirm “clear”, the muscle origin reason of the force deficits on operated side. However, the pain of knee after meniscus trauma leads to disturbances of its stability and disables the patient to load the extremity prior to surgery. These factors also limit the physical activity of patient and, finally, they can cause the fast muscle atrophy as well as the decrease of force [5]. The restoration of complete knee function after surgery lasts some weeks, especially in patients after the meniscus suture or its re-fixation. This limitation of activity increases the thigh-muscle force deficits [3, 4, 16].

The instability of knee in patients after meniscectomy can be frequently observed [12]. Disturbance in the proper pattern of knee extensors and flexors co-activation is its most frequently observed reason [17]. The optimal relation of the knee flexors (hamstrings, H) force to extensors (quadriceps, Q) force (H/Q coefficient) protects the joint against the redundant and wrongly distributed loadings at articular surfaces [18, 19]. The stability of operated knee significantly influences the stability of whole human body, the global movement coordination and, on the recurrent way, the force and endurance of thigh muscles. Studies analysing the force of knee extensors in relation to the same parameter of flexors at submaximal loadings (which are typical for many patient’s activities) confirmed this phenomenon [20–23].

The fast restoration of the proper knee function in patients after meniscectomy described in studies of Burks et al. [24] and Schimmer et al. [25] is one of reasons to call into question a necessity of introducing the supervised, postoperative rehabilitation [12, 26, 27]. On the other hand, other researchers recommended application of early rehabilitation [4, 11, 15, 16, 27]. Relatively little is known about the functional deficits in patients with the meniscus suture or its re-fixation and the possible relevance of postoperative, supervised rehabilitation [4]. Results of isokinetic muscle force examinations do not always correlate with the scores of functional assessment (one-leg rising test, one-leg-hope test) in patients treated surgically because of knee injuries [3, 28, 29].

The aim of present study is 1-year follow-up functional assessment of knee after meniscectomy or meniscus suture with subsequent rehabilitation lasting 6 months in cases of the medial meniscus tear. Measurements of knee extensors and flexors peak torques were performed for four angular velocities (60, 180, 240 and 300 s^−1^) using Biodex IV system. Moreover, the analysis of one-leg-hop and one-leg-rising clinical tests results ascertained the functional efficiency of operated knee. Results of biomechanical and clinical studies were correlated to create the objective method of treatment evaluation. We assume that the meniscus suture results in higher efficiency of operated extremity.

## Materials and methods

### Study design

Comparative, retrospective biomechanical and functional tests in healthy people and patients following two different surgeries of the torn medial meniscus were performed at the Department and Clinic for Physiotherapy, Rheumatology and Rehabilitation from 1 May 2011 to 1 July 2011 in Wiktor Dega Clinical Orthopaedic and Rehabilitation Hospital. The type of performed surgery (group A: patients after meniscectomy; group B: patients with the meniscus suture) and performing the examinations exactly 12 months from the surgery constituted independent variables. Dependent variables included peak torque of knee extensors and flexors muscles in surgically treated and non-treated extremities of patients, peak torque of knee extensors and flexors muscles in both extremities of healthy volunteers (control group) and results of functional one-leg-hop and one-leg-rising tests for both extremities in patients and healthy people.

### Subjects

Group A included 15 patients (9 women and 6 men) aged 19–35 years (25.5 ± 1.3 years on average with mean height 173 ± 8 cm and weight 68 ± 7 kg) where about 50 % of the injured posterior horn mass and/or the meniscus shaft were removed. Group B constituted 15 patients (7 women and 8 men) aged 18–38 years (23.1 ± 2.2 years on average with mean height 176 ± 9 cm and weight 70 ± 8 kg) with instable full-thickness vertical tear more than 10 mm or Bucklet handle tear type. In these patients, fasT-fix Smith and Nephew suturing system (all-inside technique) was used. Surgeries were performed by the same orthopaedist. Comparison of examinations was performed with reference to the group of healthy volunteers (control group, *N* = 15) aged 20–30 years (24.6 ± 1.1 years on average with mean height 178 ± 10 cm and weight 72 ± 6 kg). In general, they all represented similar anthropometric properties. They were recruited from personnel of Wiktor Dega Clinical Orthopaedic and Rehabilitation Hospital.

Ethical considerations were in agreement with the Helsinki Declaration. Approval was also received from the Bioethical Committee of University of Medical Sciences in Poznań (including studies on healthy people). Each patient was informed about the aim of study and gave written consent for examinations and data publication.

The primary analysis included 62 personal medical histories of patients hospitalized (surgically treated with a consequent rehabilitation) because of the isolated medial meniscus tear. After deriving them into groups depended on two main types of earlier applied surgery (meniscectomy and the meniscus suture) a clinical assessment of each patient was performed. Negative results of Steinman I and II tests, McMurray test, Lachman test and the anterior drawer sign confirming the proper function of other than meniscus knee structures were required in these patients. These examinations narrowed the recruitment to 32 patients. Time from the onset of surgical treatment ranged from 10 to 14 months (12.7 ± 2.2 months on average). Rehabilitation recommended by the leading doctor in patients from both groups lasted 4 months. It was assumed that a very good Knee Injury and Osteoarthritis Outcome Score (KOOS) [30] above 90 points was the main inclusion criteria of patients enrolled to this study. Exclusion criteria were the lack of full range of knee movement in both extremities, degenerative and inflammatory joint and spine diseases, discs herniation, central and peripheral nervous system disorders and mental disorders. During intraoperative inspections, no additional injuries of cartilages which might be considered as significant for knee function were found in both groups of patients. Meniscus surgery was the only one procedure performed in the patients. Finally, the results of biomechanical examinations and functional tests were analysed in 15 patients of group A and 15 patients of group B, because two persons abandoned this project for personal reasons. The distribution of symptomatic and asymptomatic extremities was equal in both groups of patients.

### Intervention

Patients from both groups were postoperatively treated with a supervised rehabilitation which was the same for all subjects. It aimed with diminishing the postoperative knee oedema and restoration the full range of motion and proprioception of knee. Strength and endurance of muscles in operated extremity were improved in the next step of rehabilitation. The program of rehabilitation was generally based on the neuromuscular training described by Wilk et al. [31]. The correction of the sensimotor control at the joint with its functional stability improvement was expected. Exercises in the closed kinematic chains, balance platform trainings and exercises on the stable and unstable ground with different elasticity were applied. The visual biofeedback or its deprivation was then introduced. After the full range of motion at knee was restored and no pain was reported, the programme was supplemented with exercises in the open kinematic chains as well as the running and jumping tasks on a springboard. Patients from group B did not overload the operated extremity up to 6 weeks after surgery. The orthosis limited the range of flexion up to 60º to protect the sutured meniscus which facilitated its full regeneration. Patients performed exercises 3–5 times a week under supervision of qualified physiotherapists up to reaching the full, dynamic stability of knee.

### Biomechanical examinations

The Biodex System 4 Pro dynamometer (Biodex Medical Systems, Shirley, NY, USA) for the peak torque measurements of knee extensors and flexors during concentric-isokinetic stretches was used. Dynamometer was calibrated every time before the test was applied and the patient was instructed with reference to methodology and aim of examination. Possibility of occurrence of side effects like fatigue and possible short-lasting muscle pains was explained to the patient. Warming-up with cycloergometer for 10 min was the first stage of examination.

Subjects were asked to sit on a chair and stabilized with belts crossing the chest. The additional belt stabilized the thigh at 90° of flexion in a hip joint. The axis of applicator for peak torque measurements was carefully adjusted to the knee flexion–extension axis. A free arm of applicator was mounted with a belt to the distal part of a shin, about 2–5 cm proximally from the ankle. The range of movement during a knee extension was unlimited while the flexion was restricted by the dynamometer construction and ranged maximally up to 110°. A patient performed bilaterally (non-operated extremity first) the movement of extension and flexion at the knee with the maximal force and informed about necessity of the volitional engagement. Each trial consisted of four samples with different movement repetitions at angular velocities as follows: 4 × 180, 3 × 60 , 4 × 300, 15 × 240 s^−1^. There were 15-s lasting breaks between samples for a recovery. The real value of the peak torque for the certain velocity was counted as a mean from series of movements. Obtained results were then compared to the values from studies performed with similar conditions on both sides in a group of healthy volunteers (control group). The H/Q coefficient (mean peak torque of flexors divided by mean peak torque of extensors) was additionally analysed to find out the functional stability of knee.

### Functional performance tests

Two clinical tests were supervised by one qualified physiotherapist to evaluate a function of knee. Before testing, each subject performed an active warm-up with cycloergometer for 10 min at sub-maximal level.

One-leg-hop test was described originally by Tegner et al. [32]. During this test, a subject was standing on one leg with hands behind the back and then hopped and landed, on the same leg. He could not move the hands from the back or to lose a balance. The distance was measured in centimetres from the toe in the starting position to the heel where the subject landed. The hop was repeated three times with each leg. It started with the non-operated leg. When the hop length of the subject increased in all the three performed trials then the additional hops were repeated until no increase in the hop distance was recorded. The best performance was counted from the best measurement.

One-leg-rising test was applied basing on description of Ekdahl et al. [33]. Its purpose was to assess the hip–knee extensor strength in a functional position. The subject was sitting on a chair which was easy to be adjustable for a height. The heel of one foot was placed 10 cm in front of the chair on a stool mounted on the floor. The other foot was positioned in the air straight ahead while arms were held out in front of the body. The task of a subject was to easily rise on one leg without help. The subject was allowed for three trials choosing the initial, most comfortable height of a chair. After the positive trial, the chair was lowered, and then, three new trials were allowed. The height between the height-adjustable chair and the stool the subject succeeded to rise was recorded in centimetres. The lowest value was assumed as the best result. The procedure was started from non-operated leg.

### Statistical analysis

All data were expressed as means with standard deviations values (SD). Analysis did not confirm the normal distribution of peak torque values for all applied velocities and data of both functional tests in groups A and B (Shapiro–Wilk test). To evaluate differences between results obtained in both groups of patients and healthy volunteers, the non-parametric Wilcoxon test for dependent variables was used. The value *P* ≤ 0.05 was accepted as weak significant, *P* ≤ 0.01 as moderate significant and finally *P* ≤ 0.001 as a strong significant. Parametrical Pearson’s rank correlation coefficient (*r*_p_) was used to demonstrate that the scores obtained in functional tests (one-leg-rising and one-leg-hop; separately for both groups of patients and for both lower extremities) correlate with the average peak torques of extensors and flexors muscles for all examined angular velocities (60, 180, 240, 300 s^−1^). The same levels of significance as presented above have been accepted for rank correlation studies.

## Results

The measurements of mean peak torque for muscles of both extremities in a group of healthy volunteers did not show the significant differences (Table 1). In patients after meniscectomy, the decrease in peak torque values was found particularly in the measurements from extensors and flexors groups at the low angular velocities. The most significant differences were observed in knee extensor muscles. The surgery with meniscus suture statistically changed the peak torque only in extensors muscles.

**Table 1:** Comparison of mean peak torque values (Nm) and their differences recorded with different angular velocities (s^−1^) for knee extensors and flexors from left versus right legs in healthy volunteers and operated versus non-operated legs in patients of each group

Angular velocity	Healthy volunteers *N* = 15			Group A (meniscectomy) *N* = 15			Group B (meniscus suture) *N* = 15		
	Left	Right	Difference	Operated knee	Non-operated knee	Difference	Operated knee	Non-operated knee	Difference
Knee extensors									
60	146.7	148.5	1.8	127.1	158.6	31.5***	133.2	158.5	25.3***
180	95.4	94.8	0.6	92.4	108.4	16**	95.1	91.8	3.3
240	83.9	80.8	3.1	81.3	94.5	13.2**	78.3	84.8	6.5*
300	75.6	70.9	4.7	69.1	80.5	11.4**	70.0	80.4	10.4**
Knee flexors									
60	80.9	79.1	1.8	73.8	87.5	13.7**	75.6	79.6	4.0
180	52.9	52.4	0.5	51.6	61.1	9.5**	51.8	50.2	1.6
240	51.6	55.2	3.6	51.4	56.5	5.1	50.2	50.5	0.3
300	48.8	52.8	4.0	47.0	50.5	3.5	48.3	46.7	1.6

* *P* ≤ 0.05, ** *P* ≤ 0.01, *** *P* ≤ 0.001

Comparison of the extensors mean peak torque values recorded in healthy volunteers and patients of group A showed the statistically high difference at 60 s^−1^ angular velocity and less significant result in patients of group B at the same condition (Table 2). The only difference for flexors measurements was found in patients after meniscectomy (*P* ≤ 0.05). Difference between patients from groups A and B was found only in cases of extensors peak torque measurements at the lowest angular velocity (*P* ≤ 0.05).

**Table 2:** Comparison of mean peak torque values (Nm) with different angular velocities (s^−1^) recorded from extensors and flexors from both sides in healthy volunteers and in patients from both groups (operated side only)

Angular velocity	Healthy volunteers *N* = 15	Group A (meniscectomy) *N* = 15			Group B (meniscus suture) *N* = 15	
	Mean	Mean (operated knee only)	Difference (Healthy vs. group A)	Mean (operated knee only)		Difference (Healthy vs. group B)
Knee extensors						
60	147.6	127.1#	20.5***	133.2		14.4**
180	95.1	92.4	2.7	95.1		0.0
240	82.3	81.3	1.0	78.3		4.0
300	75.6	69.1	6.5	70.0		5.6
Knee flexors						
60	80.0	73.8	6.2*	75.6		4.4
180	52.6	51.6	1.0	51.8		0.8
240	53.4	51.4	2.0	50.2		3.2
300	50.8	47.0	3.8	48.3		2.5

*P ≤ 0.05, **P ≤ 0.01, ***P ≤ 0.001

^#^Difference between A and B at * *P* ≤ 0.05

H/Q coefficients of the operated extremities differed slightly from values recorded in a group of healthy volunteers. This difference was better observed in the patients after meniscectomy at the low angular velocity 60 s^−1^ (Table 3).

**Table 3:** Comparison of H/Q coefficient with different angular velocities (s^−1^) in healthy volunteers and patients from both groups

Angular velocity	Healthy volunteers *N* = 15	Group A (meniscectomy) *N* = 15	Group B (meniscus suture) *N* = 15
	H/Q coefficient common for both sides	H/Q coefficient for operated knee	H/Q coefficient for operated knee
60	0.54	0.58*	0.56*
180	0.55	0.55	0.54
240	0.64	0.63	0.64
300	0.69	0.68	0.69

* *P* ≤ 0.05

No difference between results of functional tests for both extremities in healthy volunteers was found (Table 4). Group A patients showed a moderate difference between operated and non-operated knee while the patients with meniscus suture differed the least to the controls.

**Table 4:** Comparison of mean values of functional tests performed in healthy volunteers for both extremities and in patients of both groups for operated and non-operated side separately

Test	Healthy volunteers *N* = 15			Group A (meniscectomy) *N* = 15			Group B (meniscus suture) *N* = 15		
	Right	Left	Difference	Operated knee	Non-operated knee	Difference	Operated knee	Non-operated knee	Difference
One-leg-hop (cm)	72.5	69.5	3.0	56.5	67.5	11.0**	62.5	68.0	5.5*
One-leg- rising (*n*)	17	16	1	9	15	6**	13	16	3*

* *P* ≤ 0.05, ** *P* ≤ 0.01

*n* number of repetitions in test

There were not found correlations between results of functional tests and the peak torque measurements found for flexor muscles (Table 5). The moderate correlations were detected between the extensors mean peak torque values at 60 s^−1^ angular velocity and results of one-leg-rising test. Less significant correlations were seen in a similar fashion for one-leg-hop test.

**Table 5:** Correlation of test results between mean peak torques for knee extensor and flexor muscles measured with four different angular velocities and results of the functional tests

Angular velocity	Functional tests	Healthy volunteers *N* = 15		Patients after meniscectomy (group A) *N* = 15		Patients after meniscus suture (group B) *N* = 15	
		*r*_P_	*P*	*r*_P_	*P*	*r*_P_	*P*
Knee extensors (s^−1^)							
60	One-leg hop	0.348	0.049*	0.273	0.045*	0.302	0.043*
	One-leg rising	0.470	0.008**	0.394	0.007**	0.404	0.008**
180	One-leg hop	0.305	0.048*	0.267	0.053	0.287	0.067
	One-leg rising	0.355	0.058	0.298	0.053	0.323	0.048*
240	One-leg hop	0.222	0.052	0.226	0.064	0.198	0.053
	One-leg rising	0.265	0.068	0.251	0.057	0.247	0.059
300	One-leg hop	0.273	0.053	0.307	0.064	0.298	0.059
	One-leg rising	0.288	0.062	0.203	0.068	0.222	0.058
Knee flexors (s^−1^)							
60	One-leg hop	0.148	0.675	0.136	0.567	0.148	0.413
	One-leg rising	0.170	0.763	0.124	0.420	0.133	0.397
180	One-leg hop	0.105	0.527	0.106	0.345	0.124	0.444
	One-leg rising	0.163	0.427	0.158	0.442	0.150	0.534
240	One-leg hop	0.096	0.576	0.083	0.654	0.093	0.557
	One-leg rising	0.262	0.448	0.211	0.534	0.207	0.417
300	One-leg hop	0.120	0.437	0.097	0.584	0.093	0.486
	One-leg rising	0.299	0.358	0.256	0.337	0.282	0.478

* *P* ≤ 0.05 weak significant

** *P* ≤ 0.01 moderate significant

## Discussion

It is generally believed that the proper function of knee depends mainly on its extensors and flexors peak torque values and their mutual relationships [4]. These force values depend on the number and sequences of the proprioceptive signals transmitted from the meniscus mechanoreceptors [13]. It was found that the decrease in the muscle’s force is greater than that caused by hypotrophy taking an origin from the simple extremity immobilization after surgery [1, 5, 15]. The number of mechanoreceptors is decreased in patients after the partial meniscectomy, which can change the afferent transmission to the spinal centres both quantitatively and qualitatively. Such an “improper” neuronal transmission disturbs the function of extensors and flexors motoneurons by interneuronal connections bilaterally which finally change the muscles activity and their force during the long-lasting effort [34]. Similarly in patients after the meniscus suture the decrease in muscles force of thigh can be hypothetically explained with mainly the qualitative change of the afferent impulsation. The number of meniscus proprioceptors does not change significantly but some of them are damaged within the suture area which also changes the afferent pattern. Konishi et al. [35] using the surface electromyographical recordings showed the significant influence of the central control for thigh-muscle force generation, however, patients after the surgical reconstruction of anterior cruciate ligament were studied by them. It should be also noticed that the decrease in muscle force can produce the increase of intra-articular pressure at knee resulting in earlier development of osteoarthritis [18]. In addition, the greater compression at the knee joint surfaces (the increased intra-articular pressure) proportionally increases with the meniscus damage [4, 19]. It can be concluded that in patients with a suture of the damaged meniscus, particularly when the range of trauma is wide, there is a possibility of earlier occurrence of the degenerative changes at the operated joint, which has been documented in patients after meniscectomy [3].

The difference in peak torques values recorded from quadriceps muscles of both extremities in population of healthy people should not be >10 %. Some of researchers negate the existence of such an asymmetry if the anthropomorphic properties of examined subjects are considered [36]. In addition, the results presented in this study do not show significant differences in peak torques values recorded bilaterally in knee extensors and additionally in flexors in a group of healthy subjects (Table 1) while such a statistical difference exists in patients with 60–180 s^−1^ angular velocities, regardless of type of performed surgery (Tables 1, 2). Similar findings have been already presented, however, only in patients after the meniscectomy up to 4 years after surgery [1, 5, 11]. The decrease of peak torque for extensor muscles was also observed in researches of Gapayeva et al. [5] and Glatthorn et al. [15] at 60 s^−1^ angular velocity. Similar finding at 60 s^−1^ for knee flexors was presented in this study and has not been reported yet.

Earlier studies proved that the meniscus trauma and its subsequent surgical treatment could evoke the phenomenon of slow-contracting motor units deactivation mainly in knee extensors. The long-lasting restriction of the patient’s physical activity accompanied to the postoperative period can also evoke the additional deactivation of fast-contracting motor units [1, 15]. During our test at low angular velocity (60° s^−1^), a considerable resistance generated by dynamometer required the maximal muscle’s activation during the whole movement’s range. As a consequence, the conditions of test constrain the recruitment of majority both fast- and slow-contracting motor units [37]. When the higher angular velocities are applied (180, 240 s^−1^ and particularly 300 s^−1^), the “global” recruitment is required only at the beginning and at the end of the movement range. The fast-contracting motor units are mainly activated in the “middle phase” of performed isokinetic test [38]. Hence, in patients after the meniscus injury, the deficit of muscle force emerging at a certain contraction velocity depends mainly on the percentage of slow- and fast-contracting motor units’ activation [5]. So far, the values of extensors and flexors peak torques were referred to values recorded on the non-operated side but not to the reference values obtained in a group of healthy volunteers with a similar lifestyle, similar age, weight and height. In such a comparison, we found differences in mean values of peak torques for knee extensors between patients of both groups A and B and the healthy volunteers group (*P* ≤ 0.001; *P* ≤ 0.01) and in values of mean peak torques for knee flexors recorded in patients after meniscectomy and healthy subjects (*P* ≤ 0.05). As it can be seen in Table 2 for peak torques of extensors in patients after meniscectomy, this difference is more significant.

So far, most of the presented data in the literature regards only the knee extensors or flexors peak torques analysis separately, while little is known about mutual relationships of their actions (H/Q coefficient) [39, 40]. It seems that H/Q coefficient should provide more precise information about the stability of knee after surgery as well as the distribution of intra-articular loadings. Our study showed the existence of slight differences (*P* ≤ 0.05) in H/Q values found in two groups of examined patients (A vs. B) and between patients versus healthy volunteers. Again this phenomenon was observed at 60 s^−1^ angular velocity only.

Analysis of the functional tests results performed on both sides did not show statistically significant differences in a group of healthy volunteers (Table 4). A moderate difference (*P* ≤ 0.01) between operated and non-operated extremities in results of patients after meniscectomy was found. In patients after meniscus suture, this difference was less significant (*P* ≤ 0.05). Detected differences can be comparable to findings reported by Ericsson et al. [3], but these authors did not compare them to reference values obtained in healthy volunteers and a group of their patients was treated only with partial meniscectomy. Petsching et al. [29] and Reid et al. [41] used also one-leg-hop test for the functional evaluation of knee and their results showed asymmetry between operated and non-operated extremities. However, patients in their studies were after the reconstruction of anterior cruciate ligament. Comparing results of our functional tests, particularly to the findings of Thorstensson et al. [6] who also used one-leg-rising test, it can be expected that the osteoarthritis in operated knee appears earlier in patients after meniscectomy than meniscus repair. Stein et al. [42] also provided the evidence that arthroscopic meniscal repair significantly improved motor activity of patients and protected the osteoarthritis development when compared with partial meniscectomy. We believe that in cases where the additional significant cartilages injuries of different aetiology are found they may influence the final results of biomechanical examinations.

Our studies showed a weak correlation (*P* ≤ 0.05) between mean values of knee extensors peak torques at 60 s^−1^ angular velocity and results of one-leg-hop test (Table 5). This is opposite to the observations of Ericsson et al. [3] but this test was originally created for a functional evaluation of knee after anterior cruciate ligament reconstruction. In our study, a moderate correlation was found between mean values of knee extensors peak torques at 60 s^−1^ angular velocity and mean values of one-leg-rising test which confirms data presented by Ericsson et al. [3]. We did not notice a correlation between the mean value of knee flexors peak torque values and mean values of one-leg-rising as well as one-leg-hop tests.

Summarising the above, the existence of some correlations in results of biomechanical and clinical examinations in patients after the meniscus tear surgery with a subsequent rehabilitation imply a special usefulness of the peak torque measurements at 60 s^−1^ angular velocity and the one-leg-rising test for evaluation of a knee function. However, Östenberg et al. [36] considered the influence of age and anthropomorphic parameters of patients on such analyses which resulted in low correlations impact. One-leg-rising test seems to be particularly interesting for evaluation of knee function, probably because the subject activates all types of motor units during body-rising which produces significant overloading at low velocity. Both examinations provide similar data on quadriceps function in the operated extremity.

## Conclusions

Results of this study suggest obtaining better functional effects when meniscus suture is applied. The muscle force deficits evaluated 1 year after orthopaedic surgery oblique for consideration of modification the rehabilitative methods in a postoperative period. The particular attention should be put to the reinforcement both the quadriceps and hamstring muscles particularly after meniscectomy.
